# Cell fusion as the formation mechanism of unreduced gametes in the gynogenetic diploid hybrid fish

**DOI:** 10.1038/srep31658

**Published:** 2016-08-17

**Authors:** Jing Wang, Qingfeng Liu, Kaikun Luo, Xuan Chen, Jun Xiao, Chun Zhang, Min Tao, Rurong Zhao, Shaojun Liu

**Affiliations:** 1 Key Laboratory of Protein Chemistry and Fish Developmental Biology of Education Ministry of China, College of Life Sciences, Hunan Normal University, Changsha 410081, People’s Republic of China

## Abstract

The gynogenetic diploid hybrid clone line (GDH) derived from red crucian carp (♀ RCC) × common carp (♂ CC) possesses the unusual reproductive trait of producing unreduced diploid eggs. To identify the mechanism underlying this phenomenon, we examined the structure, *in vivo* developmental process and *in vitro* dynamic development of the GDH gonad. In summary, compared with RCC and CC, GDH showed certain special straits. First, a high frequency (84.7%) of germ cell fusion occurred in gonadal tissue culture *in vitro* as observed by time-lapse microscopy. Second, microstructural and ultrastructural observation showed numerous binucleated and multinucleated germ cells in the gonad, providing evidence of germ cell fusion *in vivo*. By contrast, in the diploid RCC and CC ovaries, neither cell fusion nor multinucleated cells were observed during the development of gonads. Third, the ovary of GDH remained at stage I for 10 months, whereas those of RCC and CC remained at that stage for 2 months, indicating that the GDH germ cells underwent abnormal development before meiosis. This report is the first to demonstrate that cell fusion facilitates the formation of unreduced gametes in vertebrates, which is a valuable finding for both evolutionary biology and reproductive biology.

Sexual organisms produce haploid gametes derived from meiosis. Fertilization of the egg by the sperm restores the chromosome number to that of the somatic cells. However, certain factors can lead to the formation of unreduced gametes, in which the genetic material is identical to that of somatic cells, in some cases specifically in females. This phenomenon is important and widespread across numerous eukaryotic taxa, including yeasts, plants, insects, amphibians, reptiles, and fish^1^,^2^,^3^,^4^. Unreduced gametes with somatic chromosome numbers represent a special germplasm in facilitating speciation and the production of new polyploid species. Several studies have suggested that the majority of polyploidization events in both plants and animals may have resulted from unreduced gamete formation^5^,^6^,^7^,^8^,^9^,^10^. This phenomenon is widely accepted as an important evolutionary force in both plants and animals. Although unreduced gametes occur spontaneously in most vertebrates, they appear to produce viable progeny mainly in ectotherms. Artificial experimentation suggests that the formation of unreduced gametes is particularly likely in fish and amphibians^11^.

There are various reasons for the formation of unreduced gametes. In fish and amphibians, the main reasons for the high frequency of unreduced gametes are that both fish and amphibians have a propensity to form reproductively successful hybrids, and they undergo external reproduction and fertilization, exposing zygotes to temperature stress, which can promote an increased production of unreduced gametes^4^. Generally, unreduced gametes are frequently produced by hybrids, facilitating polyploidization. For example, in our laboratory, the hybrid progenies from *Carassius auratus* red var. (♀) × *Cyprinus carpio* L. (♂)^12^, *Carassius auratus* red var. (♀) × *Megalobrama amblycephala* (♂)^13^, and *Carassius auratus* red var. × *Culter alburnus* (♂)^14^ can produce unreduced gametes. This phenomenon has been observed in a range of animal^4^,^15^,^16^,^17^ and plant species^18^,^19^. Interestingly, environmental stress also often triggers unreduced gamete production^20^. In response to stress, the formation of unreduced gametes may facilitate polyploid speciation and escape from genetic pressure and hostile environments.

Via interspecific hybridization producing polyploids, an allotetraploid (AT) hybrid was obtained from crossing red crucian carp (RCC; *C. auratus* red var., ♀, 2n = 100) with common carp (CC; *Cyprinus carpio* L., ♂, 2n = 100)^21^,^22^, in which both the females and males are fertile, producing diploid eggs and diploid spermatozoa, respectively. Without the treatment of doubling the chromosomes, the diploid eggs with two sets of chromosomes produced by allotetraploid hybrids developed into the first gynogenetic fish (GDH_1_, 2*n* = 100) upon activation by UV-irradiated sperm of the blunt snout bream (BSB, *Megalobrama amblycephala*). GDH_1_ was composed of entirely female diploids with 100 chromosomes. The diploid GDH_1_ presented the unique reproductive trait of producing diploid eggs. GDH_2_ fish were obtained under the same conditions as GDH_1_, and they also produced diploid eggs. The stability of producing diploid eggs provides the reproductive basis for the formation of diploid gynogenetic hybrid clonal lines. Using the same method of gynogenesis, a diploid hybrid clonal line (GDH_1_-GDH_10_) produced by gynogenesis without treatment for doubling the chromosomes was established. This diploid hybrid clonal line can produce diploid eggs for reproduction. We have undertaken various studies to research this unique reproductive characteristic, including measurement of germ cell DNA content^23^, comparison of egg size^24^, observation of the fertilization cytology^25^, research on the *Dmc1* and *cdc2/cyclin B* genes^26^, and RAPD and microsatellite analyses^27^. The results of these studies provided strong evidence for GDH stably producing diploid hybrid eggs. These previous results demonstrate the importance of further research on the mechanism of this ability of GDH.

The aim of this paper is to characterize the mechanism by which GDH produce unreduced gametes. To that end, we performed various studies, including microstructural and ultrastructral observations of gonads to analyze cell types and development and the size and shape of nuclei. To examine the gamete development process before meiosis, we developed an *ex vivo* model of the gonad to elucidate the dynamic development of the germ cell.

## Results

### Cytological characteristics of GDH gametes

**Figure 1:**
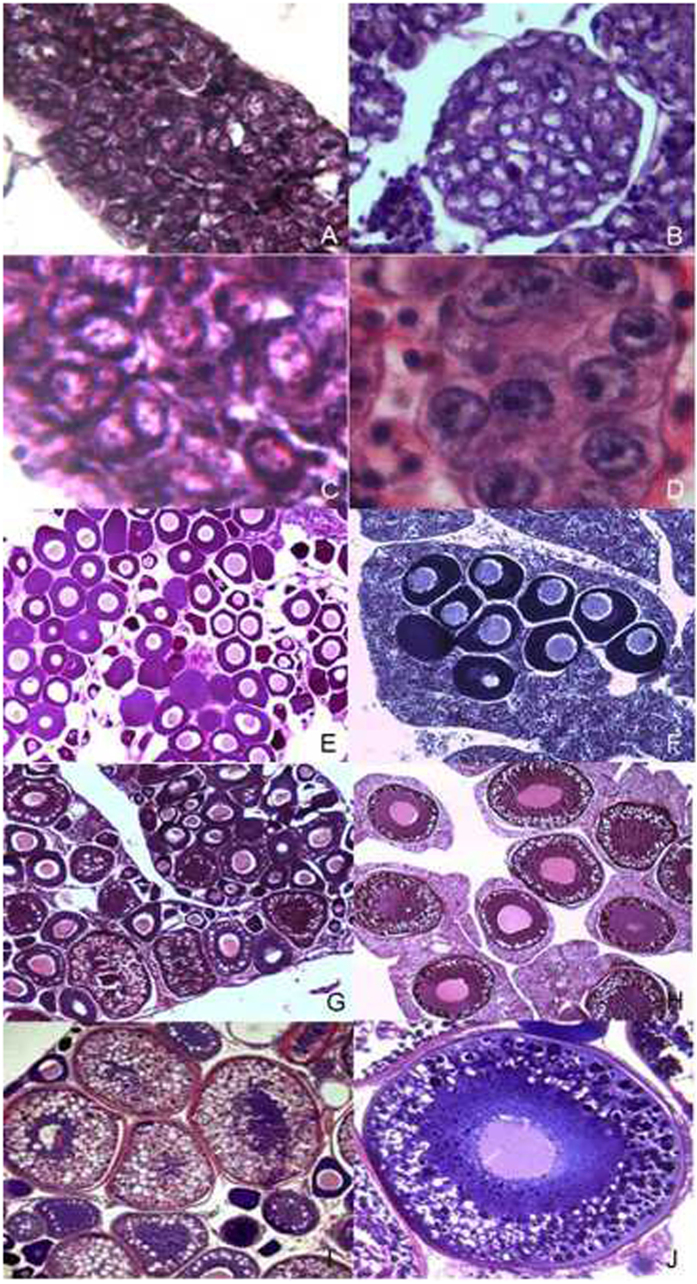
The ovarian structure of RCC and GDH. (**A,B**) Stage I ovaries of RCC and GDH, respectively (40×); (**C,D**) stage I ovaries of RCC and GDH, respectively (100×); (**E,F**) stage II ovaries of RCC and GDH, respectively (20×); (**G,H**) stage III ovaries of RCC and GDH, respectively (10×); (**I,J**) stage IV ovaries of RCC and GDH, respectively (10×).

GDH reach sexual maturity at two years old, and the control group of diploid RCC reach sexual maturity at one year old. All materials of GDH gonads were the female ovaries. Ovary development was divided into six stages according to the standards for cyprinoid fishes. Before 10 months of age, the ovary of GDH was in stage I with phase I oocytes (Fig. 1B,D). In RCC, the stage I ovarian development was shorter (before 2 months of age) (Fig. 1A,C) and quickly developed into stage II (Fig. 1E). The ovary of GDH was in stage II, containing phase I and phase II oocytes at 11–17 months of age (Fig. 1F). RCC ovaries occupied this stage at 3–4 months of age (Fig. 1E). After stage II, the gonad developed into stage III, containing oocytes of phases I, II and III (Fig. 1G,H). In stage IV, yolk sedimentation was obvious in the ovary (Fig. 1I,J), and round eggs were visible after dissection. At two years of age, the ovary of GDH was in stage V and produced mature eggs after artificially induced spawning. After that season, postnatal ovaries were in stage VI. Compared with RCC, the ovary development of GDH was slower; in particular, stage I and stage II ovaries required long development times (Table 1).

During the production season, the eggs produced by GDH were diverse in size; the first diameter class was 0.13 cm (3.47%), the same as haploid eggs of RCC; the second diameter class was 0.17 cm (93.64), the same as diploid eggs of AT; and the third diameter class was larger than 0.19 cm (2.89%), larger than diploid eggs, which may be highly polyploid eggs.

**Figure 2:**
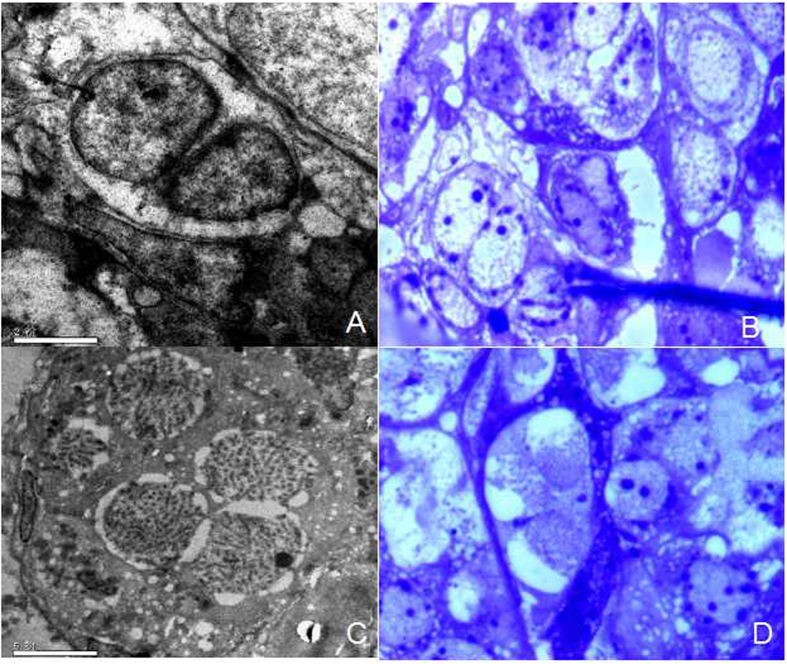
Binucleated and multinucleated cells in GDH ovary. (**A**) Binucleated cell in a 60-nm section; (**B**) binucleated cell in a 120-nm section; (**C**) multinucleated cell in a 60-nm section; (**D**) multinucleated cell in a 120 nm-section.

Notably, based on the microstructural and ultrastructural observations, there were binucleated and multinucleated cells in stage I ovaries of GDH (Fig. 2).

### Identification of cells

**Figure 3:**
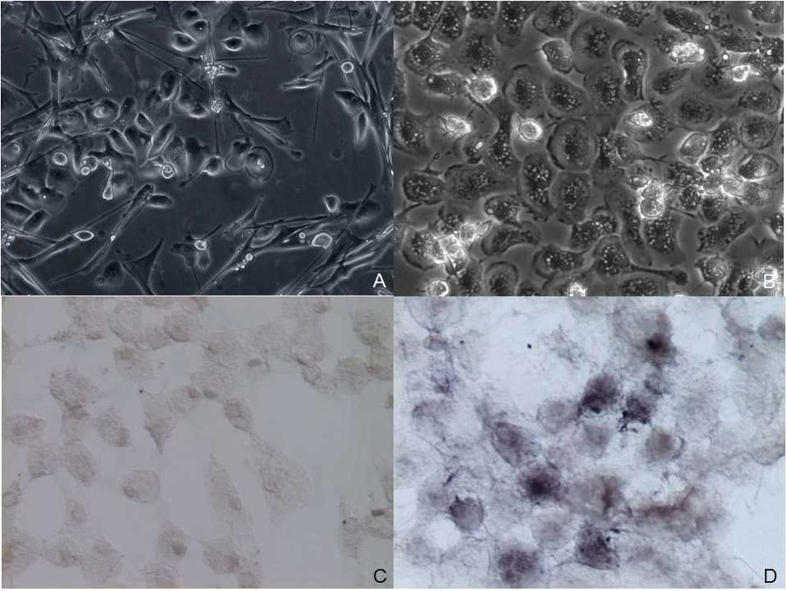
Isolation and identification of germ cells. (**A**) Fibroblasts and germ cells in ovary culture; (**B**) germ cell from isolation; (**C**) blank control for alkaline phosphatase staining; (**D**) alkaline phosphatase staining of germ cells.

**Figure 4:**
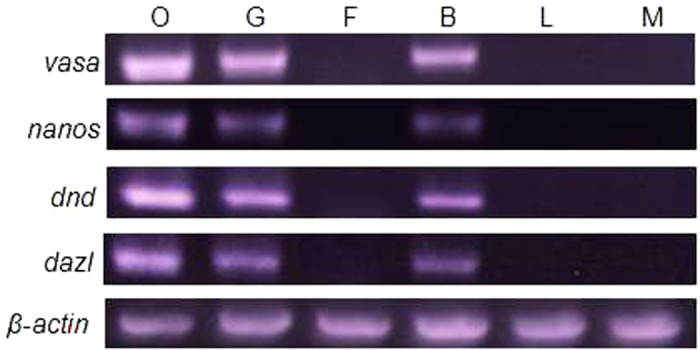
Expression of GS-specific markers by RT-PCR. Gene expression patterns of *vasa, nanos, dnd* and *dazl* in cultured cells and individual tissues. O, ovary from individual tissue; G, germ cell from ovary culture *in vitro*; F, fibroblast cell-like from ovary culture *in vitro*; L, liver from individual tissue, B, blastula; M, muscle from individual tissue.

Cells, including gametes and fibroblasts, grew from ovary tissue in primary culture (Fig. 3A). All the cultured ovaries were in stage I, and the cell type and shape did not differ among individuals. Therefore, we selected cells from 2- and 7-month-old fish for identification. Based on the differential cellular adhesion and developmental time-course properties of germ cells and fibroblasts, the cells were purified with a standard shaking method and the differential adhesion method for isolated culture. Figure 3 shows the detailed characterizations of germ cells. Germ cells were round or polygonal large, flat cells with an elevated perinuclear region (Fig. 3B). Fibroblasts were long and spindle shaped. Germ cells exhibited strong alkaline phosphatase (AP) activity (Fig. 3D) (approximately 3.0 × 10^5^ cells tested per sample). We further analyzed the germ cell identity by using the transcripts of germ cell markers of *vasa, nanos, dazl and dnd* (Fig. 4) (approximately 1.5 × 10^6^ cells collected per sample). The round cells isolated from tissue culture transcribed all four of these genes and thus appear to comprise germ cells.

### Observation of live cells

**Figure 5:**
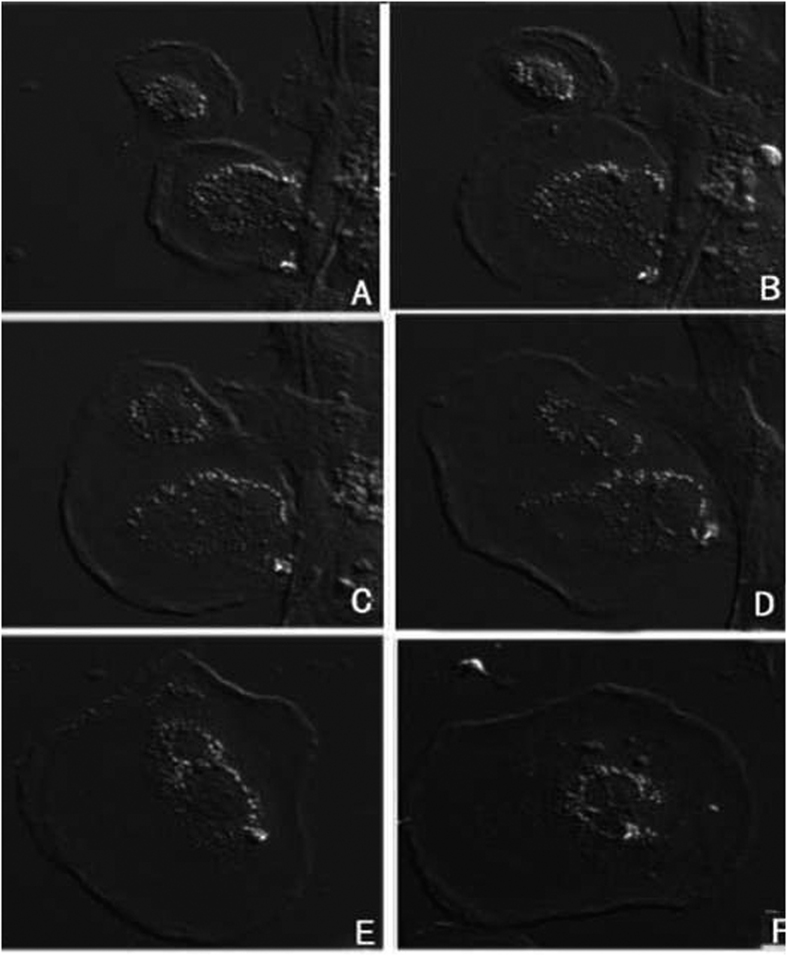
Phenomenon of cell fusion in GDH ovary (40×). (**A**) Two cells before fusion; (**B**) membranes are beginning to fuse; (**C**) cytoplasm is beginning to fuse; (**D**) the two nuclei are close to each other; (**E**) nuclei are beginning to fuse; (**F**) the two cells become a single cell with one nucleus after cell fusion.

The stage I ovary tissue of GDH and RCC grew well *in vitro* culture. Cells from both fish underwent normal mitosis. In further experiments, the germ cells in culture were photographed at 3-min intervals for 48 consecutive hours. Interestingly, the ovaries of 7-month-old GDH showed the unusual phenomenon of some cells gradually fusing (Fig. 5). In 10 samples, the cells showed fusion at a frequency of 84.7%. In the fusion process, the first step was a cell gradually moving close to another (Fig. 5A), followed by fusion of the two cell membranes (Fig. 5B) and then by the fusion of the cytoplasm (Fig. 5C); eventually, the two nuclei drew closer (Fig. 5D) until the two nuclei had fused completely (Fig. 5E), finally forming a single cell with a large nucleus (Fig. 5F). By contrast, in the RCC ovary, this gamete fusion phenomenon was not observed in *in vitro* culture (similarly, *in vitro* cultures of CC and grass carp ovaries do not show cell fusion).

## Discussion

Bisexual reproduction producing haploid gametes in vertebrates has been proven to be highly successful, and it has great importance in genetics and evolution. However, in nature, producing unreduced gametes has been considered another useful model of reproduction in evolution. The heritable genetic variation of producing unreduced gametes provides the substrate for polyploid formation. Eggs produced from GDH were diploid (unreduced gametes) based on the following evidence: 1) the eggs generated from GDH developed into the next generation (2*n* = 100) by gynogenesis without doubling the chromosomes; 2) there were tetraploids (4*n* = 200) among the progeny of GDH × AT (tetraploids) and triploids (3*n* = 150) among the progeny of GDH × CC (diploids); 3) the eggs produced by GDH were the same size as the diploid eggs generated by the allotetraploid hybrids, with a diameter of 0.17 cm^23^,^25^.

Generally, the two main factors considered to underlie the production of unreduced gametes are hybridization and environmental factors. Hybrids derived from distant hybridization (hybridization between species or higher-ranking taxa) combine two sets of chromosomes from different taxa. In meiosis, the hybrids are unable to form the normal bivalent pairings because only homologous chromosomes can pair up. Therefore, the progeny derived from hybridization replicate the genome to undergo meiosis and produce diploid gametes. Notably, environmental stress may facilitate the formation of unreduced gametes; for example, changes to the temperature-sensitive proteins involved in meiosis may result in the formation of unreduced gametes. In addition to the above causes, certain other factors also prompt the formation of unreduced gametes; in fish and amphibians, unreduced gametes are commonly induced by factors such as irradiation, chemical treatment, pressure, and cold or heat shock^4^. The formation of unreduced gametes is likely to be retained in species lineages through specific gametes to prevent species extinction. Thus, unreduced gametes can be considered as a factor driving speciation.

The prevalence of unreduced gametes is involved in crosses between species and in crosses between ploidy levels. High levels of unreduced gamete production by hybrid fish and amphibians are well documented^4^.

In this paper, the reason unreduced eggs were produced in GDH was distant hybridization. GDH originated from a distant hybridization of RCC and CC, which belong to different genera. Via gynogenesis, the hybrid diploid eggs from allotetraploid hybrids formed all-female GDH clonal lines containing the chromosomes of two different species. For reproduction, it was difficult for the hybrid diploid GDH to undergo normal meiosis. Instead, these hybrids must perform a unique reproduction mode involving the duplication of the genome before meiosis and production of diploid gametes.

The frequency of producing unreduced gametes differs among species. Humans produce unreduced sperm at a frequency of 0.73%^28^, and triploid embryos are observed in mice^29^ and chickens^30^ at frequencies of 0.59% and 0.43%, respectively. In GDH, unreduced gametes were observed at the high frequency of above 93%. In addition, with increasing generations, the proportion of diploid eggs produced by GDH also increased.

**Figure 6:**
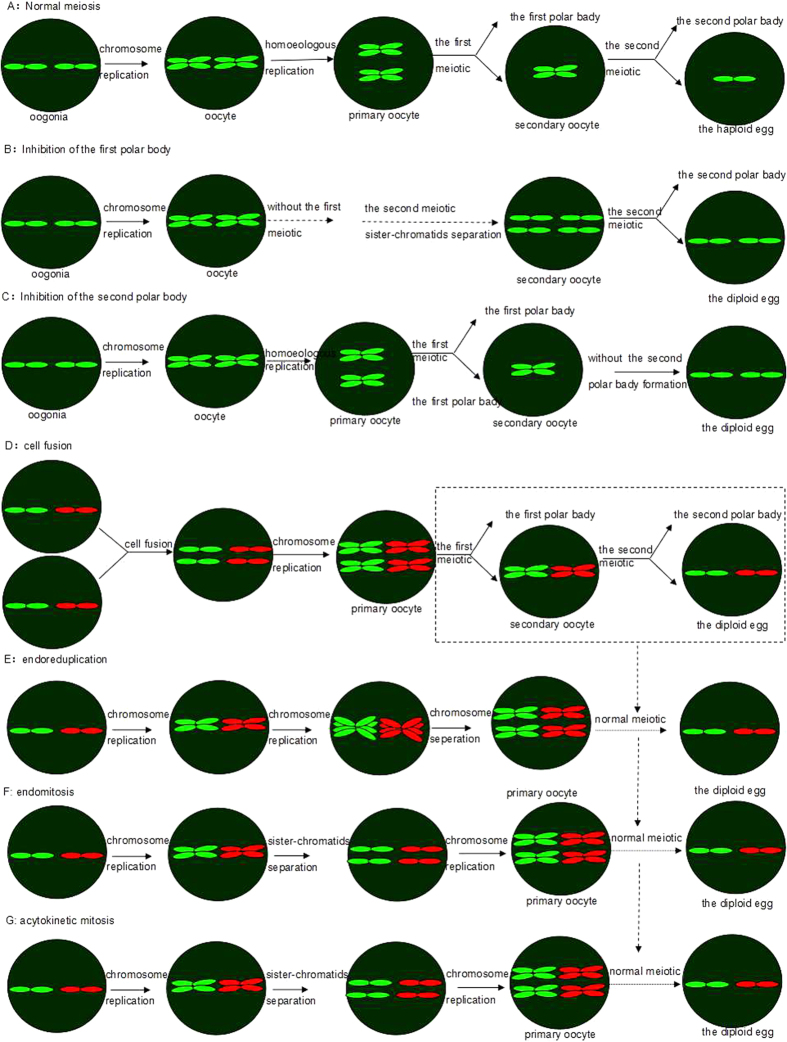
Model of the meiotic process producing unreduced gametes. Schematics present the cytological mechanisms for unreduced egg formation. In normal meiosis, a single round of DNA replication is followed by two consecutive divisions, resulting in a haploid egg and two polar bodies (**A**). Inhibition of the release of the first or second polar body produces an unreduced diploid egg and one polar body (**B,C**). Chromosomes of two species are shown in red and in green. Premeiotic doubling of chromosomes gives rise to hybridogenetic diploid eggs, including melding two G0-phase cells into a single cell containing a 4N nuclei (**D**, cell fusion); by arresting cells in G2-phase and then inducing another S-phase producing diplochromosomes (individual centromeres with four chromatids) (**E**, endoreduplication); by arresting cells in M-phase (M*) in the absence of the nuclear membrane and also without the formation of the spindle, producing a giant nucleus (4N) in a cell (**F**, endomitosis); or by completing karyokinesis in the absence of cytokinesis, producing two nuclei in a single cell [**G**, acytokinetic mitosis]. After doubling chromosomes, the gametes undergo normal meiosis and produce unreduced eggs.

Unreduced gamete production is present across widely disparate phyla^16^,^18^ and occurs via a plethora of different mechanisms^18^,^31^,^32^. This phenomenon most commonly arises through meiotic defects. At present, there are several hypotheses regarding the mechanism of the production of unreduced gametes: first, inhibition of the first meiotic division (Fig. 6B); second, preventing the second meiotic division (Fig. 6C); third, doubling the genetic material before meiosis (Fig. 6D–G). Meiosis is a specialized cell division that is essential for sexual reproduction. The normal meiotic process includes one-time chromosome duplication, formation of bivalent cells, and subsequently two successive divisions to produce cells with half the chromosome number of the mother cell (Fig. 6A). In the first meiotic division, meiosis I, the homologous chromosomes are separated in what is referred to as a reductional division. Meiosis II resembles mitosis in that it involves the separation of sister chromatids and is referred to as an equational division.

The formation of unreduced gametes results from abnormal meiosis. For example, in flowering plants, unreduced gametes have been observed to result from a very wide range of mechanisms, including premeiotic doubling of chromosome number, complete loss of the first or second meiotic division, and defects in meiotic cell plate formation, spindle orientation, or cytokinesis^31^,^32^,^33^. Some green algae species appear to produce unreduced gametes through abnormal spindle orientations or failed cytokinesis^34^. In asexually reproducing diploid stick insects, the fusion of two haploid nuclei produces unreduced gametes that subsequently develop into embryos^35^. In some fish and amphibians, unreduced gametes are often formed by spindle misdivision with subsequent retention of a secondary polar body^4^. In addition, chromosome doubling (two rounds of endo-reduplication instead of one) or fusion of two nuclei before meiosis is known to produce unreduced gametes in some parthenogenetic lizards and fish^36^. Another common mechanism for polyploid formation in fish and amphibians, inhibition of the first mitotic division after fertilization^4^, may arguably fall outside the scope of unreduced gamete production because it does not occur during meiosis, but it can be classed as a related phenomenon.

Oogenesis in GDH is basically the same as in other cyprinid fishes: primary oocytes undergo meiotic division to yield secondary oocytes and the first polar bodies; activation of the egg by the sperm stimulates the second reduction division of the secondary oocyte (most fish eggs are arrested in metaphase II). GDH is a hybrid clone line, and the chromosomes of this line derive from both RCC and CC. RCC and CC belong to different genera, and their crossing is therefore considered distant hybridization. We know that during normal meiotic prophase I, the meiosis-specific events of pairing and recombination between homologous chromosomes occur. These processes are important not only for generating genetic variability in the offspring but also for establishing the attachments between chromosomes that are required for the subsequent divisions. However, the chromosomes in GDH could not properly pair during meiosis. Therefore, the potential mechanism of inhibition of releasing the second polar body (Fig. 6C) could not occur in GDH. Observation of the fertilization cytology of GDH indicated that the second polar body was exhausted normally under the effect of fertilization, ruling out the inhibition of the second meiotic division^25^. Notably, if the reason was the absence of the first meiosis (Fig. 6B), the egg size of GDH would be close to that of the haploid eggs produced from diploid because retaining the first polar body does not markedly change the egg size, and the diameter of GDH eggs would be 0.13 cm rather than 0.17 cm. Thus, the premeiotic replication could overcome the production of unreduced gametes in GDH. The chromosome numbers in GDH oogonia were 100 (2*n*), 200 (4*n*) and 380 (close to 8*n*) during metaphase^23^, which provides some support for the mechanism of premeiotic replication.

Generally, unreduced gametes are prevalent in diverse plant and animal species and occur via a wide range of molecular and cytogenetic mechanisms^4^,^31^,^32^. Previous reviews have focused on the conditions under which unreduced gametes form^31^ as well as their ecological significance and occurrence^18^. Recently, the molecular and cytological mechanisms of unreduced gamete formation have been under study^32^,^33^.

One well-studied genetic pathway that can lead to unreduced gametes is the premeiotic replication of chromosomes to obtain an additional copy of the genome without cytokinesis at the beginning of gametogenesis. In vertebrates, premeiotic doubling of chromosomes to produce unreduced gametes has been documented in lizards and loaches^36^.

Zakir Ullah^37^ proposed four mechanisms for the nuclear duplication: first, completing mitosis in the absence of cytokinesis [acytokinetic mitosis, G_1_→S→G_2_→M(c^−^)→G_1_]; second, melding two G_0_-phase cells into a single cell containing two G-phase nuclei (cell fusion, G_1_→S→G_2_→M→G_0_→fusion); third, arresting cells in G_2_-phase and then undergoing another S-phase (endoreduplication, G_1_→S→G_2_→S→G); fourth, arresting cells in M-phase (M*) in the absence of cytokinesis (endomitosis, G_1_→S→G_2_→M*→S→G→M*). Multiple rounds of acytokinetic mitosis produce multinucleated cells (Fig. 6G). Multiple rounds of endoreduplication (endocycles) produce mononucleated cells (Fig. 6E), whereas multiple rounds of endomitosis produce a single multilobular nucleus (Fig. 6F). Endomitosis is similar to endoreduplication. Whereas endoreduplication results from arresting cells in G_2_-phase, before they enter mitosis, endomitosis results from arresting cells within M-phase, before they complete mitosis. The premeiotic doubling of chromosomes allows bivalent formation to occur as in the diploid hybrids without homologous chromosomes in a cell.

Unreduced gametes produced by a premeiotic endomitotic chromosome doubling are observed in some species^38^,^39^. Premeiotic endoreduplication results in preferential pairing, with synapsis likely involving a recombination between doubled sister chromatids over homolog chromosomes.

Previous studies have indicated that the production of diploid eggs by GDH is associated with nuclear duplication in the early stage of germ cell development, before meiosis^23^, ruling out the inhibition of the production of the first or second polar body.

Observation of gonad development *in vivo* indicated that GDH reached sexual maturity at two years old, whereas the allotetraploid hybrids from RCC × CC reached sexual maturity at one year old. The main reason for this difference was that the ovaries of GDH remained in stage I for 10 months, markedly longer than that of RCC and allotetraploid hybrids. Perhaps the oogonia in stage I underwent an abnormal development process to duplicate the genome.

Multinucleated cells can arise by cell fusion, a process in which G_0_-phase cells simply fuse their membranes together, to produce a single cell in which a giant nucleus is distributed in the cytoplasm. In GDH, cell fusion was a frequent rather than a rare event. Recent reports have suggested that cell fusion may explain some of the observed plasticity of adult stem cells^40^,^41^,^42^. The reports examined spontaneous fusion between embryonic stem cells and either unfractionated bone marrow cells^41^, neural stem cells, or differentiated neural cells^42^. In those reports, the frequency of cell fusion was low^41^,^42^. In germ cells, cell fusion may be considered as part of meiosis. The best way to observe cell fusion is via live cell imaging using time-lapse microscopy. The gonad culture system described here provides an *ex vivo* tool with which to examine the process of the early stage of gonadal development.

Cell fusion occurred in the ovary of GDH after 6 months of age. Before this time, only mitosis was observed in the cultured ovarian tissue. Data from time-lapse microscopy experiments also indicated a high frequency of cell fusion. Thus, GDH could produce numerous diploid eggs. Although the cell fusion of adult stem cells and germ cell have the same effect, their mechanisms differ.

GDH derived from RCC × CC produced unreduced gametes, whereas the tetraploid hybrids derived from RCC × CC produced reduced gametes. The main difference in reproduction between GDH and the tetraploid hybrids is the ploidy of germ cells at the beginning of gametogenesis, with GDH cells each containing one copy of RCC chromosomes and CC chromosomes and AT cells each containing two copies. Nonhomologous chromosomes in diploid hybrids are generally considered difficult to pair and form bivalents, probably due to the poor affinities between them. Thus, chromosome pairing was likely the major problem in hybrid reproduction and was the driving force for unreduced gametes. Because GDH is a hybrid diploid, it contains a set of RCC chromosomes and a set of CC chromosomes. During meiosis, the chromosome pairing is disordered, unable to develop into physiologically normal eggs. For the species to continue, the gametes require a periodic adjustment to form another set of chromosomes, providing the germ cell with the normal meiotic chromosomes in homoeologous pairing. In GDH, nonhomologous chromosomes do not pair and thus are doubled by premeiotic fusion in germ cells. Cell fusion duplicates all the chromosomes in the germ cell. Before cell fusion, there are only nonhomologous chromosomes in the germ cell; after cell fusion, there are two identical sets of chromosomes. This condition allows the chromosomes to form bivalent pairs and then behave like a normal diploid individual producing reduced gametes.

Another tetraploid hybrid was obtained from the fertilization of diploid eggs from GDH with diploid sperm of male hormonally sex-reversed GDH, which never produced unreduced tetraploid gametes, only diploid eggs. Such a process suggests that the production of unreduced gametes is not regulated by the presence or absence of specific gene(s) but instead by a mechanism that checks the ability to pair germ cell chromosomes. If the unreduced gametogenesis is not controlled by a combination of chromosomes but, rather, by a hypothetical specific major gene, unreduced tetraploid gametes could be formed in the tetraploids. This phenomenon also occurs in *Misgurnus* loaches^43^.

The results of the present study provide the first evidence for the mechanism by which unreduced gametes result from cell fusion in vertebrates. Further studies will be required to resolve several of the questions raised by these observations, such as the frequency of the fusion and the timing of cell fusion during ovary development *in vivo*.

## Methods

### Ethics

All samples, including gynogenetic diploid hybrids (GDH), red crucian carp (RCC) and common carp (CC), were cultured in ponds at the Protection Station of Polyploidy Fish, Hunan Normal University, and fed with artificial feed. All experiments were approved by the Animal Care Committee of Hunan Normal University and followed the guidelines of the Administration of Affairs Concerning Animal Experimentation of China. Fish were deeply anesthetized with 100 mg/l MS-222 (Sigma-Aldrich, St Louis, MO, USA) before dissection.

### Overview of the procedure

**Figure 7:**
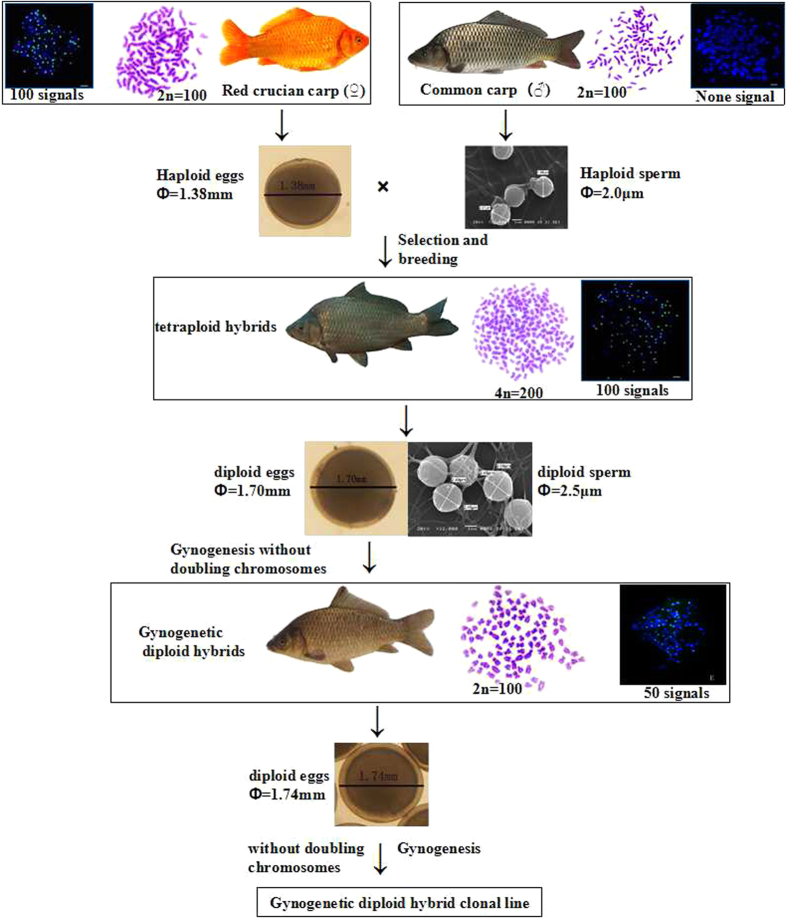
Formation of the gynogenetic diploid hybrid clone line. Schematic represents the production of allotetraploids (AT) and gynogenetic diploid hybrids (GDH) and their reproductive systems. Fertilization of haploid eggs of RCC with haploid sperm of CC produces AT. Gynogenesis of diploid eggs from AT without chromosome doubling produced the first generation of GDH (GDH_1_). The eggs produced from GDH_1_ were 1.74 mm in diameter, close to size of the diploid eggs of AT, which were 1.70 mm in diameter. Using the same method of gynogenesis without chromosome doubling, successive generations were obtained (GDH_2_-GDH_10_). In this figure, the FISH signals indicate the number of crucian carp chromosomes in the samples.

Figure 7 illustrates the procedure by which the gynogenetic diploid hybrid clonal line was developed. The first generation of gynogenetic offspring (GDH_1_) (1997) was obtained by artificial gynogenesis from the eggs of AT that were activated with ultraviolet (UV)-treated sterilized sperm of blunt snout bream without treatment for doubling the chromosomes. During the reproductive seasons (April to June) of 1999–2015, nine generations were obtained, and a gynogenetic diploid hybrid clonal line (GDH_1_–GDH_10_) was formed in succession. Under artificial breeding, the fry were initially cultured in ponds (approximately 40 m^2^) at a density of 200 fish/m^2^, and once they had reached 3.5–4.0 cm in length, they were transferred to larger ponds (approximately 667 m^2^) at a density of 10 fish/m^2^ for continued growth. During the reproductive season (water temperature at 20–22 °C), the parental fish were cultured in round ponds (approximately 120 m^2^) at a density of 5 fish/m^2^ for spawning.

### Observation of the gonadal structure

To observe gonadal structures, 10 sample individuals were selected every month from birth to two years old. The gonads were fixed in 2.5% glutaraldehyde solution, washed with phosphate buffer, transferred into osmic acid solution, dehydrated in a graded acetone series, and finally embedded in Epon 812. Ultrathin sections were cut; 60-nm sections were stained with uranyl acetate and lead citrate and imaged using a JEOL-1230 (Japan) electron microscope, and 120-nm sections were stained with toluidine blue and imaged using a Pixera Pro 600ES microscope. Some of the gonads were instead fixed in Bouin’s solution for the preparation of paraffin-embedded tissue. Sections were then cut and stained with hematoxylin and eosin. Microstructure was observed and photographed using a Pixera Pro 600ES microscope. During the reproductive season, the diameters of 1.0 × 10^5^ mature eggs from 10 samples were measured.

### Cell culture and identification

The above sample gonads from GDH before 10 months of age and RCC before 2 months of age were also used for vitro culture. After anesthesia and blood sampling, tissue samples were sterilized with 70% ethanol. Gonads were dissected and minced into 1-mm^3^ pieces before being washed and resuspended. The fragments were seeded in 35-mm dishes and incubated for 1 h. Then, the tissue masses were cultured (26 °C, 5% CO_2_) in Dulbecco’s modified Eagle’s medium (Gibco BRL, Burlington, ON) containing 5 ng/ml bFGF (Gibco BRL), 15% FBS (Hyclone Labs Inc., Logan, UT), 100 U/ml penicillin, 100 μg/ml streptomycin and supplemented with 5% heat-inactivated fish serum. Tissue fragments were removed after one week, when new cells had grown from tissue fragments. Cell identification via alkaline phosphatase and RT-PCR to check the expression of gametes cell-specific markers were performed as described^44^. The gene-specific primer information is provided in Table 2.

### Time-lapse microscopy

To observe the developmental dynamics of germ cells, time-lapse imaging was performed using a Nikon Ti-E microscope for all the cultured samples. Cells were seeded in 35-mm glass-bottom culture dishes (MatTek Corporation) 24 h before imaging. An image was recorded every 3 min for a continuous period of approximately 48 h (approximately 1.5 × 10^3^ cells were observed from each fish).

## Additional Information

**How to cite this article**: Wang, J. *et al*. Cell fusion as the formation mechanism of unreduced gametes in the gynogenetic diploid hybrid fish. *Sci. Rep.* **6**, 31658; doi: 10.1038/srep31658 (2016).

## Figures and Tables

**Table 1:** Comparison of ovary development between RCC and GDH.

Type	Age (months)	1–2	3–4	5–6	7–10	11–13	14–17	18–19	20–23	24–25
RCC	Stage of ovary development	I	II	III	IV	V				
	Sample size (cm)	5.8 ± 1.0	9.1 ± 1.3	16.7 ± 2.3	20.5 ± 3.2	22.7 ± 3.8				
GDH	Stage of ovary development	I	I	I	I	II	II	III	IV	V
	Sample size (cm)	5.2 ± 0.7	8.5 ± 1.1	15.3 ± 2.1	18.7 ± 2.4	19.8 ± 2.8	21.2 ± 3.1	22.4 ± 3.5	23.8 ± 3.2	25.1 ± 3.8

**Table 2:** Primers and PCR conditions.

Gene	Primer sequences	Ta (°C)	Product size (bp)
*vasa*	F-AAGAGGCTTTGGTAGAGGAGGTT R-TGGACAGGAGTAGGCTTCACATA	50	324
*nanos*	F-GCAACCCATCCGAGAAGAAG R-TCCACGAGCGGGCAGAAT	54	190
*dazl*	F-GAAAAAACTCAAACTGGGACCT R-GGCACCTGTGGGACAATAAAT	52	225
*dnd*	F-CCAGAGCATTGGCACTATTT R-CAGACATCATTCGCAGCAT	51	254
